# Investigation of leptin and leptin receptor gene polymorphisms in schizophrenia: further support for an association with attempted suicide

**DOI:** 10.1017/neu.2026.10058

**Published:** 2026-02-10

**Authors:** Hasan Mervan Aytac, Yasemin Oyaci, Eren Aytac, Mustafa Pehlivan, Huseyin Sehit Burhan, Furkan Bahadir Alptekin, Sacide Pehlivan

**Affiliations:** 1 Institute of Graduate Studies in Health Sciences, Istanbul University, Istanbul, Türkiye; 2 Department of Psychiatry, Basaksehir Cam and Sakura City Hospital, University of Health Sciences https://ror.org/05grcz969r , Istanbul, Türkiye; 3 Department of Basic Sciences, Istanbul Nisantasi University, Istanbul, Türkiye; 4 Psychiatry Unit, Varto State Hospital, Mus, Türkiye; 5 Department of Hematology, Basaksehir Cam and Sakura City Hospital, Istanbul, Türkiye; 6 Department of Medical Biology, Istanbul Faculty of Medicine, Istanbul University, Türkiye

**Keywords:** schizophrenia, leptin, leptin receptor, single nucleotide polymorphism, suicide attempt

## Abstract

**Objective:**

This study aimed to investigate leptin (*LEP*) (*G*-2548A) and leptin receptor (*LEPR*) (668A>G) gene polymorphisms in schizophrenia patients with and without suicide attempts, compared to controls.

**Methods:**

The study included 120 patients with schizophrenia and 130 healthy volunteers. Sociodemographic characteristics, suicidal behavior, and symptom severity were assessed using data collection forms. Gene polymorphisms were analyzed from DNA samples using the polymerase chain reaction–restriction fragment length polymorphism.

**Results:**

The *LEP* genotype distribution in schizophrenia patients differed significantly from controls, with the heterozygous GA genotype more frequent in controls (*p* = 0.026). Within schizophrenia, *LEPR* genotype distribution differed by suicide attempt history; the heterozygous AG genotype was more frequent in non-attempters (*p* = 0.048). Logistic regression showed that the *LEPR* polymorphism (*p* = 0.023), number of hospitalizations (*p* = 0.036), and Positive and Negative Syndrome Scale-psychopathology score (*p* = 0.023) predict suicide attempt history in schizophrenia.

**Conclusion:**

Our findings suggest that *LEP* polymorphism may contribute to schizophrenia susceptibility, while *LEPR* polymorphism may be linked to suicide attempts in schizophrenia.

Significant Outcomes

- The distribution of the leptin *(LEP)* genotype differed significantly between schizophrenia (SCZ) patients and healthy controls, with the heterozygous *LEP* GA genotype being more prevalent in controls than in SCZ patients (*p* = 0.026).
- Among SCZ patients, the distribution of leptin receptor *(LEPR)* genotypes varied significantly according to suicide attempt history, with the heterozygous *LEPR* AG genotype being more common in non-attempted patients than in those with suicide attempts (*p* = 0.048).
- Logistic regression analysis indicated that *LEPR* gene polymorphism (*p* = 0.023), number of hospitalizations (*p* = 0.036), and Positive and Negative Syndrome Scale-psychopathology scores (*p* = 0.023) were significant independent predictors of suicide attempt history in SCZ patients.

Limitations

- Due to timing constraints, serum leptin levels could not be measured alongside the genetic analysis of *LEP* and *LEPR* polymorphisms.
- The relatively small sample size may have reduced the statistical power of the findings, limiting the generalizability of the results.
- The cross-sectional nature of the study may limit causal inferences about *LEP* and *LEPR* gene polymorphisms in SCZ.

## Introduction

Schizophrenia (SCZ) is a chronic and severe psychiatric disorder associated with a markedly increased risk of suicide. Lifetime suicide rates among individuals with SCZ are estimated to range between 4% and 10%, while suicide attempts occur in up to half of affected patients (Hor & Taylor, 2010; Olfson *et al*., 2021; Correll *et al*., 2022). This heightened vulnerability is driven by a complex interaction of demographic, clinical, psychosocial, and biological factors. Clinically, depressive symptoms, prior suicide attempts, substance misuse, hopelessness, poor treatment adherence, and recent hospital discharge have consistently been identified as robust predictors of suicidal behavior in SCZ. Demographic characteristics, including younger age, male sex, higher education, and good premorbid functioning, have also been linked to increased suicide risk (Hawton *et al*., 2005; Carlborg *et al*., 2010; Hor & Taylor, 2010; Sher & Kahn, 2019; Li *et al*., 2025). In addition, psychosocial stressors such as social isolation, family history of psychiatric disorders or suicide, and childhood trauma, particularly sexual abuse and physical neglect, further increase vulnerability to suicidal behavior in individuals with SCZ. (Mohammadzadeh *et al*., 2019). Beyond these established risk factors, emerging evidence underscores the contribution of genetic mechanisms – including specific single nucleotide polymorphisms (e.g., in *SIRT1*, *MTHFR, ACP1,* complement component *C4, TNF-α, MBL2*, and *NR3C1*) (Li *et al*., 2017; Aytac *et al*., 2020; Liu *et al*., 2020; Aytac *et al*., 2022; Uçak *et al*., 2022, Ebrahimi *et al*., 2024; Oyaci *et al*., 2024), altered gene expression in glial and immune-related genes (*ALDH1L1*, *GS*, *CX3CR1*, *P2RY12*) (Zhang *et al*., 2020) in the prefrontal cortex, and broader polygenic influences – in shaping suicide risk in SCZ. Together, evidence from demographic, clinical, psychosocial, and genetic studies highlights the multifactorial nature of suicidal behavior in individuals with SCZ and underscores the need for integrated clinical and genetic approaches to risk assessment and prevention (Pehlivan *et al*., 2022; Goker *et al*., 2023; Oyaci *et al*., 2023; Ozdilli *et al*., 2024).

Among the biological systems proposed to link metabolic regulation with neuropsychiatric outcomes, the leptin signalling pathway has received increasing attention. Leptin is a pleiotropic hormone involved not only in energy homeostasis but also in neurodevelopment, synaptic plasticity, stress responsivity, and emotional regulation (Park & Ahima, 2015). The leptin gene (*LEP*), composed of three exons, is located on human chromosome 7q32.1 (Isse *et al*., 1995). Recent large-scale genetic studies have identified a locus near 7q32.1 on chromosome 7 as significantly associated with suicide attempts. This link appears to be independent of psychiatric diagnoses, implying a direct genetic contribution to suicide risk (Fiori *et al*., 2025). *LEP* is highly polymorphic, with numerous single nucleotide polymorphisms (SNPs) found throughout its promoter, exon, and intron regions. A notable variant, (-2548G>A) (rs7799039), involves a guanine-to-adenine substitution at position -2,548 upstream of the ATG start codon in the *LEP* promoter (Mammès *et al*., 1998).

Leptin exerts its biological effects through binding to its receptor, which activates intracellular signalling pathways that increase energy expenditure and reduce nutrient intake. Leptin receptor belongs to the cytokine receptor superfamily and is expressed in various tissues, including the placenta. The leptin receptor gene (*LEPR*) is situated on chromosome 1 at cytogenetic band 1p31.3 (Marroqui *et al*., 2012; Aytac *et al*., 2025). Large-scale genetic analyses have also implicated a locus on chromosome 1 in suicide attempt risk, though results are somewhat inconsistent, establishing chromosome 1 as a key region in the genetic architecture of suicide (Kimbrel *et al*., 2022). SNPs within *LEPR* have been extensively studied and may impair receptor signalling by favoring the production of the truncated Ob-Ra isoform over the full-length Ob-Rb, or by decreasing receptor expression on the cell surface, thereby limiting leptin receptor interactions. One important SNP, rs1137101 (*LEPR* c.668A>G), has been shown to affect leptin receptor structure and function (Saad *et al*., 2020).

Leptin has also been extensively investigated in SCZ through genetic, epigenetic, and clinical perspectives. Several *LEP* gene polymorphisms (e.g., rs3828942 and c.-2548G/A) have been associated with an increased risk of metabolic syndrome, although their direct link to SCZ remains uncertain (Boiko *et al*., 2022). Epigenetic studies show increased DNA methylation in the *LEP* promoter and reduced leptin expression in patients, with methylation at specific CpG sites inversely correlated with positive symptom severity (Song *et al*., 2022). Clinically, serum leptin levels are generally elevated in SCZ patients even after adjusting for body mass index (BMI) and medication use, correlating with metabolic syndrome; however, some subgroups exhibit lower leptin levels, indicating biological heterogeneity (Akan *et al*., 2013; Stubbs *et al*., 2016; Petrikis *et al*., 2022). Mendelian randomisation studies do not support a causal role for leptin levels in SCZ risk, but leptin/adiponectin ratios and correlations with metabolic markers highlight its biomarker potential (Chen *et al*., 2018b, 2021). Lower leptin levels have been linked to greater severity of suicidal behavior in SCZ spectrum disorders, and leptin receptor polymorphisms (e.g., rs1171276) have been associated with increased suicide risk in other psychiatric populations, such as depression, potentially through mechanisms involving leptin resistance and impulsivity (Acikel *et al*., 2020b). While leptin and its receptor have been implicated in both SCZ and suicidal behavior through altered circulating levels, gene expression, and genetic variants, evidence on their specific role in suicide risk among SCZ patients is limited. To date, no studies have specifically examined leptin gene polymorphisms in relation to suicide attempts in SCZ.

Based on the existing evidence, we hypothesised that *LEP* (G-2548A) and *LEPR* (668A>G) polymorphisms may be involved in suicide attempt risk among patients with SCZ. Accordingly, the present study aimed to compare *LEP* and *LEPR* genotype distributions between SCZ patients and healthy controls, and to investigate the relationship between these polymorphisms and suicide attempts within the SCZ group.

## Method

### Patient selection

This case-control study included 120 patients diagnosed with SCZ who were consecutively recruited over a three-month period from the outpatient psychiatry clinic of Malazgirt State Hospital, along with 130 healthy control participants matched for age, sex, BMI, and ethnicity.

#### Inclusion criteria

For the patient group, inclusion criteria were a DSM-IV diagnosis of SCZ confirmed by the Structured Clinical Interview for DSM-IV Axis I Disorders (SCID-I), age between 18 and 65 years, literacy, and absence of intellectual disability or neurodevelopmental disorders.

#### Exclusion criteria

Exclusion criteria for the patient group included refusal to participate, the presence of any Axis I psychiatric disorder other than SCZ according to SCID-I, SCZ secondary to substance use disorders or general medical conditions, and the presence of neurological or systemic diseases that could impair cognitive functioning.

The healthy control group consisted of volunteers recruited from hospital staff working at the same institution and within the same geographic region as the patient group. All control participants underwent a detailed clinical interview conducted by a psychiatrist to exclude current or lifetime psychiatric disorders according to DSM-IV criteria. Individuals with a personal history of suicide attempts, non-suicidal self-injury, substance use disorders, neurodevelopmental disorders, or major neurological or systemic medical conditions were excluded from the control group. In addition, participants with a first-degree family history of SCZ or other psychotic disorders were not included. These procedures were implemented to ensure that the control group represented a psychiatrically healthy population and to minimise potential confounding factors in genetic comparisons.

## Diagnostic tools and scales

Participants were informed about the study’s aim, materials, and methods, and written informed consent was obtained. Sociodemographic data, clinical parameters related to SCZ, and information regarding suicide attempts, methods of suicide, type of suicide, and family history of suicide were collected. In addition, a detailed clinical information interview form prepared by the researchers was administered. Subsequently, the Structured Clinical Interview for DSM-IV Axis I Disorders (SCID-I) was conducted to confirm the patients’ diagnoses (First *et al*., 1997, Çorapçıoğlu *et al*., 1999). For participants with SCZ, the Positive and Negative Syndrome Scale (PANSS) (Kay *et al*., 1987, Kostakoğlu *et al*., 1999) and the Clinical Global Impression Scale (CGI) were used to assess symptom severity (Guy, 1976).

### DNA analyses

Genotyping of *LEP* -2548G/A (rs7799039) and *LEPR* 668A/G (rs1137101) single nucleotide polymorphisms (SNPs) was performed using the polymerase chain reaction–restriction fragment length polymorphism (PCR–RFLP) method. PCR amplification was carried out with the following primer sequences: forward 5’-TTTCCTGTAATTTTCCCGTGAG-3’ and reverse 5’-AAAGCAAAAGACAGGCATAAAAA-3’ for *LEP* -2548G/A, and forward 5’-GCCTAATCCAGTATTTTATATCTG-3’ and reverse 5’-GCCACTCTTAATACCCCCCAGTAC-3’ for *LEPR* 668A/G.

For the *LEP* -2548G>A polymorphism, genotyping was performed using the HhaI restriction enzyme, while the *LEPR* 668A>G polymorphism was analyzed with the MspI enzyme. Following agarose gel electrophoresis, the samples were visualised under UV light, and genotypes were determined. The fragment patterns were as follows: for *LEP* -2548G>A, GG: 242 bp; GA: 242, 181, and 61 bp; AA: 181 and 61 bp. For *LEPR* 668A>G, AA: 416 bp; AG: 416, 229, and 187 bp; GG: 229 and 187 bp (Aytac *et al*., 2025).

### Statistical analyses

Statistical analyses were performed using IBM SPSS Statistics for Windows, version 21.0. Descriptive data were presented as means ± standard deviations for continuous variables and as frequencies and percentages for categorical variables. Continuous variables were assessed for normality using visual inspection of histograms and *Q*–*Q* plots together with the Shapiro–Wilk test, and homogeneity of variances was evaluated using Levene’s test. Normally distributed continuous variables are presented as mean ± standard deviation and were compared between groups using independent samples *t*-tests; Welch’s *t*-test was applied when the assumption of homogeneity of variances was violated. Non-normally distributed continuous variables are presented as median (interquartile range) and were compared using the Mann–Whitney *U* test. Group comparisons for categorical variables were conducted using Pearson’s chi-square or Fisher’s exact test.

Hardy–Weinberg equilibrium (HWE) was tested using an online calculator (https://gene-calc.pl/hardy-weinberg-page), and all polymorphisms conformed to HWE (*χ* ^2^ test, *p* > 0.05) in both SCZ and control groups. Post hoc power analysis was conducted using G*Power version 3.0.5 (http://www.psycho.uni-duesseldorf.de/abteilungen/aap/gpower3/) with a goodness-of-fit *χ* ^2^ test, assuming an alpha error probability of 0.05. Potential population stratification bias was evaluated according to the method described by Lee and Wang. At the time of study design and sample collection, the minimum required sample size was calculated as 200 participants (100 per group) to achieve at least 80% statistical power at *α* = 0.05.

Demographic, clinical, and genetic variables associated with suicide attempt history were first examined using univariate logistic regression analyses, and variables with a *p* value <0.10 were subsequently entered into a multivariate logistic regression model to identify independent predictors, with results expressed as adjusted odds ratios and 95% confidence intervals. Model adequacy was assessed using standard goodness-of-fit indices, and multicollinearity was evaluated using variance inflation factors. All statistical tests were two-tailed, and a *p* value <0.05 was considered statistically significant.

## Results

### LEP and LEPR genotyping

A total of 120 patients with SCZ (33 females, 87 males) and 130 age- and sex-matched healthy controls were evaluated in terms of their clinical characteristics and psychometric scale scores (Tables 1 and 2). There was no significant difference in *LEPR* genotype distribution between patients with SCZ and healthy controls. In contrast, the *LEP* genotype distribution differed significantly between the two groups. Specifically, the frequency of the heterozygous GA genotype was significantly higher in the healthy control group compared to the SCZ group (OR: 1.767; 95% Cl: 1.068–2.925; *p* = 0.026; GA vs. AA + GG) (Table 3). Interaction analysis of *LEP* and *LEPR* gene genotypes revealed that none were associated with a significantly increased risk of SCZ (Table 4).

**Table 1.** Clinical scale scores of patients with SCZ

	SCZ patients (*N*: 120)	Healthy controls (*N*: 130)
	Mean ± SD	Mean ± SD
**Age**	40.88 ± 10.60	43.90 ± 16.37
**Age of onset (year)**	24.85 ± 8.38	
**Duration of disorder (year)**	16.06 ± 9.63	
**Number of hospitalisation**	3.51 ± 4.69	
**PANSS-pos.**	11.72 ± 3.92	
**PANSS-neg.**	16.00 ± 5.41	
**PANSS-psycho.**	29.98 ± 7.37	
**PANSS-total**	57.71 ± 13.26	
**CGI-S**	5.34 ± 0.83	
**CGI-I**	2.23 ± 0.77	

SCZ, schizophrenia; PANSS, positive and negative syndrome scale; CGI-S, clinical global impression-severity; CGI-I, the clinical global impression-improvement; pos, positive; neg, negative; psycho, psychopathology.

**Table 2.** Sociodemographic and clinical characteristics of patients with SCZ

		*N*	%
**Sex**	**Female**	33	27.5
	**Male**	87	72.5
**Education**	**Literate**	10	8.3
	**Primary School**	49	40.8
	**Secondary School**	20	16.7
	**High School**	32	26.7
	**University**	9	7.5
**Marital status**	**Single**	81	67.5
	**Married**	29	24.2
	**Widow**	2	1.7
	**Divorced**	8	6.7
**Working status**	**Unemployed**	67	55.8
	**Housewife**	28	23.3
	**Officer/employee**	14	11.7
	**Tradesman**	3	2.5
	**Retired**	8	6.7
**Physical illness**	**No**	86	71.7
	**Yes**	34	28.3
**Surgery**	**No**	88	73.3
	**Yes**	32	26.7
**Cigarette**	**No**	51	42.5
	**Yes**	69	57.5
**Suicide attempt**	**No**	85	70.8
	**Yes**	35	29.2
**Methods of suicide**	**Poisoning**	22	62.8
	**Jumping from a height**	9	25.7
	**Cutting or piercing**	3	8.6
	**Hanging**	1	2.9
**Type of suicide**	**Violent**	13	37.2
	**Non-violent**	22	62.8
**Family history of suicide**	**No**	115	95.8
	**Yes**	5	4.2

**Table 3.** Comparison of *LEP* (-2548G>A) and *LEPR* (668A>G) polymorphism frequencies between patients with SCZ and healthy controls

		SCZ	Healthy control			
	Genotype	*n* = 120 (%)	*n* = 130 (%)	OR	95% CI	*p*
***–2548G>A***						
Recessive	AA	17 (14.2)	12 (9.2)	0.616*	0.281–1.351*	0.223*
Over-dominant	GA	58 (48.3)	81 (62.3)	1.767*	1.068–2.925*	0.026*
Dominant	GG	45 (37.5)	37 (28.5)	0.663*	0.390–1.127*	0.128*
Additive	AA vs. GG			1.165*	0.494–2.746*	0.727*
**Allele**				**OR**		
	A	92 (38.3)	105 (40.4)			
	G	148 (61.7)	155 (59.6)	1.090*	0.761–1.561*	0.639*
***668A>G***						
Dominant	AA	89 (74.2)	91 (70)	0.813*	0.467–1.415*	0.464*
Over-dominant	AG	28 (23.3)	32 (24.6)	1.073*	0.600–1.919*	0.813*
Recessive	GG	3 (2.5)	7 (5.4)	2.220^&^	0.561–8.787^&^	0.338^&^
Additive	AA vs. GG			2.282^&^	0.572–9.105^&^	0.333^&^
**Allele**				**OR**		
	A	206 (85.8)	214 (82.3)			
	G	34 (14.2)	46 (17.7)	0.768*	0.474–1.244*	0.283*

^*^; Pearson chi-square, ^&^; Fisher’s Exact Test, SCZ; schizophrenia, OR; odds ratio, CI; confidence interval.

*LEP* (-2548G>A): Recessive, AA vs. GA+GG; Over-dominant, GA vs. AA + GG; Dominant, GG vs. AA+GA.

*LEPR* (668A>G): Dominant, AA vs. AG+GG; Over-dominant, AG vs. AA + GG; Recessive, GG vs. AA+AG.

**Table 4.** Interaction analysis of *LEP* (-2548G>A) and *LEPR* (668A>G) polymorphisms in SCZ risk

-2548G>A	668A>G	SCZ (%)	Control (%)	OR (95% CI)	*p* value
AA	AA	13 (10.8)	10 (7.7)	0.686 (0.289–1.628)*	0.391*
AA	AG	4 (3.3)	2 (1.5)	1.51 (0.08 –2.520)^&^	0.431^&^
AA	GG	0 (0.0)	0 (0.0)	–	–
GA	AA	41 (34.2)	55 (42.3)	1.413 (0.846–2.361)*	0.186*
GA	AG	15 (12.5)	21 (16.2)	1.349 (0.660–2.756)*	0.411*
GA	GG	2 (1.7)	5 (3.8)	2.360 (0.449–12.400)^&^	0.449^&^
GG	AA	35 (29.2)	26 (20)	0.607 (0.339–1.087)*	0.092*
GG	AG	9 (7.5)	9 (6.9)	0.917 (0.352–2.394)*	0.860*
GG	GG	1 (0.8)	2 (1.5)	1.859 (0.166–20.773)^&^	1.000^&^

SCZ; schizophrenia, OR; odd ratio, CI; confident interval, *; Pearson chi-square, &; Fisher’s exact test.

### Comparison of LEP and LEPR genotype and allele frequency distributions in SCZ patients with and without a history of suicide attempt

Analysis of *LEPR* genotype distributions in SCZ patients, based on the presence or absence of a suicide attempt history, revealed significant differences between those with and without a history of suicide attempts. Specifically, the AG genotype frequencies were notably higher in the group without a history of suicide attempt (OR: 0.328; 95% CI: 0.105–1.029; *p* = 0.048; AG vs. AA + GG). However, *LEP* genotype and allele frequency distributions did not show statistically significant differences between those with and without a history of suicide attempt (*p* > 0.005) (Table 5).

**Table 5.** Comparison of *LEP* (−2548G>A) and *LEPR* (668A>G) polymorphism frequencies between SCZ patients with and without a history of suicide attempt

		Attempted suicide				
	Genotype	Yes *n* = 35 (%)	No *n* = 85 (%)	OR	95% CI	p*
***-2548G>A***						
Recessive	AA	3 (8.6)	14 (16.5)	2.103^&^	0.565–7.834^&^	0.389^&^
Over-dominant	GA	17 (48.6)	41 (48.2)	0.987*	0.449–2.169*	0.973*
Dominant	GG	15 (42.9)	30 (35.3)	0.727*	0.326–1.625*	0.437*
Additive	AA vs. GG			1.480^&^	1.184–1850^&^	0.348^&^
**Allele**				**OR**		
	A	23 (32.9)	69 (40.6)			
	G	47 (67.1)	101 (59.4)	0.429*	0.106–1.725*	0.263*
***668A>G***						
Dominant	AA	29 (82.9)	60 (70.6)	0.497*	0.184–1.343*	0.163*
Over-dominant	AG	4 (11.4)	24 (28.2)	0.328*	0.105–1.029*	0.048*
Recessive	GG	2 (5.7)	1 (1.2)	0.196^&^	0.017–2.240^&^	0.203^&^
Additive	AA vs. GG			0.242^&^	0.021–2.776^&^	0.262^&^
**Allele**				**OR**		
	A	62 (88.6)	144 (84.7)			
	G	8 (11.4)	26 (15.3)	.715*	0.307–1.666*	0.435*

^*^; Pearson chi-square, ^&^; Fisher’s Exact Test, OR; odds ratio, CI; confidence interval.

*LEP* (-2548G>A): Recessive, AA vs. GA+GG; Over-dominant, GA vs. AA + GG; Dominant, GG vs. AA+GA.

*LEPR* (668A>G): Dominant, AA vs. AG+GG; Over-dominant, AG vs. AA + GG; Recessive, GG vs. AA+AG.

### Comparison of demographic and clinical variables according to suicide attempt history

Demographic and clinical characteristics of patients with SCZ were compared according to the presence or absence of a history of suicide attempt (Table 6). Patients with a history of suicide attempt were significantly older than those without such a history (*p* = 0.040) and had a significantly longer duration of disorder (*p* = 0.014). No significant difference was observed between the groups with respect to age at illness onset (*p* = 0.923).

**Table 6.** Comparison of demographic and clinical variables according to suicide attempt history

	Attempted suicide			
	Yes (*n* = 35)	No (*n* = 85)	Test	*p* value
**Age (years)**	43.97 ± 9.58	39.61 ± 10.80	t	**0.040**
**Duration of disorder (years)**	19.43 ± 9.21	14.68 ± 9.51	t	**0.014**
**Age of onset (years)**	24.74 ± 7.54	24.91 ± 8.75	t	0.923
**CGI-S**	5.63 ± 0.60	5.22 ± 0.89	†	**0.005**
**CGI-I**	2.43 ± 0.65	2.15 ± 0.81	t	0.076
**PANSS-pos.**	12.29 ± 3.95	11.49 ± 3.92	t	0.318
**PANSS-neg.**	16.94 ± 3.98	15.62 ± 5.88	t	0.226
**PANSS-psycho.**	32.86 ± 7.37	28.80 ± 7.08	t	**0.006**
**Number of hospitalisation**	4 (2–6)	2 (1–4)	MWU	**0.001**

t, Independent samples *t*-test; MWU, Mann–Whitney *U* test; †, Welch’s *t*-test (*t*-test with unequal variances). PANSS, positive and negative syndrome scale; CGI-S, clinical global impression-severity; CGI-I, the clinical global impression-improvement; pos, positive; neg, negative; psyho, psychopathology.

Regarding clinical severity, patients with a history of suicide attempt exhibited significantly higher CGI–Severity scores compared with patients without a suicide attempt history (*p* = 0.005). However, CGI–Improvement scores did not differ significantly between the two groups (*p* = 0.076). In terms of psychopathology, PANSS-psychopathology scores were significantly higher in patients with a history of suicide attempt (*p* = 0.006). By contrast, PANSS positive (*p* = 0.318) and negative (*p* = 0.226) subscale scores did not differ significantly between the groups. Finally, patients with a history of suicide attempt had a significantly higher number of hospitalisations compared with those without a suicide attempt history (*p* = 0.001).

### Multiple logistic regression analysis of factors associated with suicide attempt history in patients with SCZ

In the univariate logistic regression analysis, a history of suicide attempt was used as the dependent variable. All demographic, clinical, and genetic variables were initially evaluated in univariate analyses. Variables with a *p* value less than 0.1 in the univariate analysis were included in the multivariate logistic regression model: age (OR: 1.040; 95% CI: 1.001–1.081; *p* = 0.044), *LEPR* gene polymorphism (OR: 3.049; 95% CI: 0.972–9.566; *p* = 0.056), number of hospitalisations (OR: 1.120; 95% CI: 1.023–1.225; *p* = 0.014), duration of disorder (OR: 1.053; 95% CI: 1.009–1.098; *p* = 0.017), PANSS-psychopathology score (OR: 1.078; 95% CI: 1.019–1.139; *p* = 0.009), CGI-S score (OR: 1.898; 95% CI: 1.116–3.227; *p* = 0.018), and CGI-I score (OR: 1.595; 95% CI: 0.947–2.687; *p* = 0.079). In the multivariate logistic regression analysis, *LEPR* gene polymorphism (OR: 4.597; 95% CI: 1.232–17.152*; p* = 0.023), number of hospitalisations (OR: 1.105; 95% CI: 1.006–1.213; *p* = 0.036), and PANSS-psychopathology score (OR: 1.097; 95% CI: 1.013–1.188; *p* = 0.023) were identified as independent predictors of suicide attempt history. Results are presented in Table 7.

**Table 7.** Univariate and multivariate logistic regression analyses for suicide attempt history in patients with SCZ

	Univariate		Multivariate	
	OR (95% CI)	*p*	OR (95% CI)	*p*
**Sex**	1.406 (0.562–3.517)	0.466		
**Age**	1.040 (1.001–1.081)	0.044*	1.007 (0.950–1.068)	0.803
***LEP* (-2548G>A)**	1.375 (0.615–3.072)	0.437		
***LEPR* (668A>G)**	3.049 (0.972–9.566)	0.056*	4.597 (1.232–17.152)	**0.023**
**Number of hospitalisation**	1.120 (1.023–1.225)	0.014*	1.105 (1.006–1.213)	**0.036**
**Duration of disorder**	1.053 (1.009–1.098)	0.017*	1.018 (0.956–1.085)	0.580
**Age of onset**	0.998 (0.951–1.046)	0.923		
** PANSS-pos.**	1.051 (0.954–1.158)	0.317		
** PANSS-neg.**	1.045 (0.973–1.123)	0.227		
**PANSS-psycho.**	1.078 (1.019–1.139)	0.009*	1.097 (1.013–1.188)	**0.023**
**CGI-S**	1.898 (1.116–3.227)	0.018*	1.565 (0.814–3.011)	0.179
**CGI-I**	1.595 (0.947–2.687)	0.079*	0.731 (0.355–1.505)	0.396

Initial –2 Log Likelihood: 144.874; Final –2 Log Likelihood: 120.332; Omnibus Test: *χ* ^2^ = 24.541, *p* = 0.001; Nagelkerke *R* ^2^ = 0.264; Hosmer–Lemeshow Test: χ^2^ = 4.498, *p* = 0.810. LEP, leptin gene; LEPR, leptin receptor gene; PANSS, positive and negative syndrome scale; CGI-S, clinical global impression-severity; CGI-I, the clinical global impression-improvement; pos, positive; neg, negative; psycho, psychopathology. * Variables with a *p* value < 0.10 in univariate analyses were included in the multivariate logistic regression model.

## Discussion

Our findings revealed that the *LEP* genotype distribution differed significantly between SCZ patients and healthy individuals. Although the overall *LEPR* genotype distribution did not differ significantly between the SCZ and control groups, subgroup analyses based on suicide attempt history within the patient group demonstrated significant differences in *LEPR* genotype distribution. Although the relevant literature indicates that *LEP* and *LEPR* gene polymorphisms have been extensively investigated in SCZ – mainly in relation to their association with metabolic side effects rather than as direct risk factors for the disorder – there are very few studies that directly examine whether the *LEP* or *LEPR* polymorphisms are genetically implicated in patients diagnosed with SCZ. While Chen et al., found that drug-naïve SCZ patients exhibited some incidence of metabolic disorders, polymorphisms in the *LEP* (-2548G/A) and *5-HTR2C* (-759C/T) genes were not associated with SCZ or metabolic disorders (Chen *et al*., 2018a). Akan et al., reported that the frequency of genotypes carrying the A allele of the *LEP* -2548G/A polymorphism (GA + AA), was two-fold lower in SCZ patients compared to controls, and no significant difference was observed in *LEPR* p.Q223R genotypes (Akan *et al*., 2013). In our study, consistent with the findings of Akan *et al*., the frequency of the heterozygous GA genotype was significantly higher in the healthy control group compared to the SCZ group. We speculate that heterozygosity in the *LEPR* gene may confer a protective advantage against the development of SCZ.

A large meta-analysis reported that leptin levels are modestly to moderately elevated in individuals with SCZ compared to controls, particularly among females and those with multiple episodes of illness. This elevation remains significant even after adjusting for BMI and antipsychotic medication use, indicating that illness-related factors and lifestyle may also contribute (Stubbs *et al*., 2016). Numerous studies have confirmed increased leptin levels in both early and chronic phases of SCZ, as well as in patients treated with second-generation antipsychotics (Potvin *et al*., 2015; Martorell *et al*., 2019). However, some research has found no significant differences or even reduced fasting leptin levels in certain subgroups, reflecting variability likely due to differences in sample characteristics and study design (Nurjono *et al*., 2014; Erzin *et al*., 2018; Martorell *et al*., 2019). Considering that *LEP* and *LEPR* gene polymorphisms can influence leptin expression and receptor functionality, the higher frequency of the heterozygous GA genotype observed in healthy controls compared to patients with SCZ may reflect a genetic profile associated with relatively balanced leptin signalling. Such heterozygosity could plausibly help maintain leptin activity within an optimal physiological range, thereby avoiding both insufficient and excessive leptin signalling, which have been implicated in metabolic dysregulation, altered stress responsivity, and neurobiological vulnerability in SCZ.

Analysis of *LEPR* genotype distributions among SCZ patients, stratified by suicide attempt history, revealed significant differences between those with and without such a history. Furthermore, multivariate logistic regression identified *LEPR* gene polymorphism as a significant predictor of suicide attempts. Notably, the frequency of the AG genotype was significantly higher in SCZ patients without a history of suicide attempts. Currently, no studies directly investigate *LEP* or *LEPR* gene polymorphisms in SCZ patients with a history of suicide attempts. While one study reported that the *LEPR* rs1171276 polymorphism is associated with higher suicide probability scores in depressed adolescents, suggesting a potential link between leptin signalling and suicidal behavior in psychiatric populations, this association has not been specifically examined in SCZ or with the more commonly studied *LEPR* variants (Acikel *et al*., 2020a). Research on suicide risk in SCZ has primarily focused on other genes, such as *NR3C1* (glucocorticoid receptor), *5-HTTLPR* (serotonin transporter), *HTR2A*, *MIF*, and *MBL2*, some of which have shown associations with suicide attempts (de Medeiros Alves *et al*., 2015; Bani-Fatemi *et al*., 2016; Aytac *et al*., 2020; Oyaci *et al*., 2024). These findings underscore the complex genetic landscape underlying suicide risk in SCZ.

A 2020 meta-analysis of nine case-control studies reported significantly lower serum leptin levels in individuals exhibiting suicidal behavior compared to controls, suggesting a potential association between low leptin and heightened suicide risk (González-Castro *et al*., 2020). Several investigations involving psychiatric patients – including those with SCZ, major depressive disorder, and bipolar disorder – have found that suicide attempters exhibit lower serum or cerebrospinal fluid (CSF) leptin levels relative to healthy controls (Atmaca *et al*., 2002, 2003, 2008). Notably, women with major depressive disorder who attempted suicide showed reduced CSF leptin compared to women with other diagnoses, implying a possible gender-specific effect (Westling *et al*., 2004). Additionally, decreased leptin levels were more pronounced in violent suicide attempters compared to non-violent attempters and controls (Atmaca *et al*., 2008). Conversely, some studies reported no significant differences in leptin levels between suicide attempters and non-attempters after adjusting for confounders such as BMI, suggesting that other metabolic or clinical factors may modulate this relationship (da Graça Cantarelli *et al*., 2015, Papadopoulou *et al*., 2017).

The leptin receptor plays a crucial role in regulating energy balance and appetite through leptin signalling. The 668A>G polymorphism influences receptor functionality, potentially affecting leptin binding or signal transduction. Specifically, the G allele (coding for arginine) is linked to reduced receptor sensitivity or altered signalling, which may result in elevated circulating leptin levels due to compensatory responses to diminished receptor activity (Hryachkova *et al*., 2024). For example, in women with preeclampsia or pregnancy-induced hypertension, carriers of the G668 allele (either AG or GG genotypes) exhibit significantly higher leptin levels compared to normotensive controls, suggesting that this polymorphism can increase leptin in certain pathological conditions (Tennekoon *et al*., 2012). In our study, we observed a higher prevalence of the heterozygous AG genotype among non-suicidal SCZ patients, implying that this genotype may be less common in those with a history of suicide attempts. This pattern suggests that the homozygous AA or GG genotypes could be more frequent in suicidal patients. The heterozygous AG state might represent a balanced leptin signalling profile, avoiding the extremes of receptor dysfunction seen in GG-associated leptin resistance or AA-associated receptor overactivity, thereby potentially contributing to mood stabilisation or reduced impulsivity in SCZ patients.

In our study, the number of hospitalisations and the PANSS-psychopathology scores were identified as independent predictors of suicide attempt history, alongside *LEPR* gene polymorphism, in multivariate logistic regression analysis. This finding aligns with extensive existing research showing that both the frequency of psychiatric hospitalisations and elevated PANSS scores serve as independent predictors of suicide attempts in individuals with SCZ. Multiple large-scale meta-analyses and cohort studies have demonstrated that a higher number of hospitalisations reflects greater illness severity, treatment resistance, or recurrent psychiatric crises, all of which increase suicide risk (Cassidy *et al*., 2018; Zaheer *et al*., 2020; Mamtani *et al*., 2024). Similarly, elevated PANSS general psychopathology scores–which encompass symptoms such as depression, anxiety, and cognitive impairment—have been consistently associated with an increased likelihood of suicide attempts (Cassidy *et al*., 2018; Pascariu *et al*., 2022, Fernández *et al*., 2024). Taken together, our findings reinforce the critical role of clinical severity markers, alongside genetic factors like *LEPR* polymorphisms, in shaping suicide risk profiles in SCZ patients.

One of the key strengths of our study is its novel focus on investigating *LEP* (-2548G>A) and *LEPR* (668A>G) gene polymorphisms specifically in SCZ patients with a history of suicide attempts, comparing them to both SCZ patients without suicide attempts and healthy controls. This comprehensive comparison enhances the clinical relevance of our findings. Additionally, recruiting all participants from the same ethnic group and geographic region helped minimise environmental and population stratification biases, thereby strengthening the internal validity of the results. Although our study did not include epigenetic analyses, this homogeneity in sampling provides a robust genetic investigation framework. Furthermore, our use of peripheral blood samples facilitates less invasive and more feasible genetic testing, which is practical for clinical research.

Despite these strengths, there are important limitations to consider. The relatively small sample size may have limited the statistical power to detect subtle genetic associations, reducing the generalizability of the findings. The cross-sectional design restricts our ability to infer causality between *LEP* and *LEPR* polymorphisms and SCZ or suicide risk. Moreover, the lack of serum leptin measurements prevents direct correlation of genotypes with leptin levels, limiting functional interpretation. Another limitation is that our genetic analyses were conducted on peripheral blood samples rather than central nervous system tissues or CSF, which might more directly reflect neurobiological mechanisms underlying SCZ and suicidal behavior.

In summary, our findings suggest that the *LEP* gene polymorphism may contribute to susceptibility to SCZ in the Turkish population, whereas the *LEPR* gene polymorphism appears to be associated with suicide attempts among individuals with SCZ. Moreover, logistic regression analysis indicated that the *LEPR* polymorphism, number of hospitalisations, and PANSS-psychopathology score were significant predictors of suicide attempt history in patients with SCZ. From a clinical perspective, these results highlight the potential relevance of leptin pathway–related genetic variation for understanding biological heterogeneity in SCZ and for informing future suicide risk stratification approaches when integrated with established clinical indicators. Although these findings do not have immediate diagnostic or therapeutic implications, they provide valuable preliminary insights into the genetic factors underlying suicide risk in SCZ. Importantly, future research should aim to replicate these findings in larger and more diverse samples using longitudinal designs, and to integrate genetic data with biochemical, epigenetic, and central nervous system–derived measures, which will be essential for deepening our understanding and validating the observed associations.

## Data Availability

Data is available from the corresponding author upon reasonable request.
